# Potential of Pléiades and WorldView-3 Tri-Stereo DSMs to Represent Heights of Small Isolated Objects

**DOI:** 10.3390/s20092695

**Published:** 2020-05-09

**Authors:** Ana-Maria Loghin, Johannes Otepka-Schremmer, Norbert Pfeifer

**Affiliations:** Department of Geodesy and Geoinformation, Technische Universität Wien, Wiedner Hauptstraße 8-10, 1040 Vienna, Austria; Johannes.Otepka@geo.tuwien.ac.at (J.O.-S.); Norbert.Pfeifer@geo.tuwien.ac.at (N.P.)

**Keywords:** VHR tri-stereo satellite imagery, digital elevation model, isolated objects, dense image matching

## Abstract

High-resolution stereo and multi-view imagery are used for digital surface model (DSM) derivation over large areas for numerous applications in topography, cartography, geomorphology, and 3D surface modelling. Dense image matching is a key component in 3D reconstruction and mapping, although the 3D reconstruction process encounters difficulties for water surfaces, areas with no texture or with a repetitive pattern appearance in the images, and for very small objects. This study investigates the capabilities and limitations of space-borne very high resolution imagery, specifically Pléiades (0.70 m) and WorldView-3 (0.31 m) imagery, with respect to the automatic point cloud reconstruction of small isolated objects. For this purpose, single buildings, vehicles, and trees were analyzed. The main focus is to quantify their detectability in the photogrammetrically-derived DSMs by estimating their heights as a function of object type and size. The estimated height was investigated with respect to the following parameters: building length and width, vehicle length and width, and tree crown diameter. Manually measured object heights from the oriented images were used as a reference. We demonstrate that the DSM-based estimated height of a single object strongly depends on its size, and we quantify this effect. Starting from very small objects, which are not elevated against their surroundings, and ending with large objects, we obtained a gradual increase of the relative heights. For small vehicles, buildings, and trees (lengths <7 pixels, crown diameters <4 pixels), the Pléiades-derived DSM showed less than 20% or none of the actual object’s height. For large vehicles, buildings, and trees (lengths >14 pixels, crown diameters >7 pixels), the estimated heights were higher than 60% of the real values. In the case of the WorldView-3 derived DSM, the estimated height of small vehicles, buildings, and trees (lengths <16 pixels, crown diameters <8 pixels) was less than 50% of their actual height, whereas larger objects (lengths >33 pixels, crown diameters >16 pixels) were reconstructed at more than 90% in height.

## 1. Introduction

For more than thirty years, civilian satellite sensors have been used for digital elevation model (DEM) extraction over large areas in a timely and cost-effective manner for a wide range of applications in engineering, land planning, and environmental management. Beginning with the year 1986, when SPOT—the first satellite providing stereo-images, with a panchromatic Ground Sampling Distance (GSD) of 10 m—was launched [1], the optical satellite industry has been experiencing continuous development. Today more and more space sensors are available that acquire not only stereo but also tri-stereo satellite imagery. The generation of high and very high resolution commercial space imaging systems for DEM generation started in September 1999, with the launch of IKONOS [2]. Among the Very High Resolution (VHR) optical satellites providing along- and across-track stereo, the following systems can be mentioned: Ziyuan-3 (2.1 m), KOMPSAT-2 (1 m), Gaofen-2 (0.8 m), TripleSat (0.8 m), EROS B (0.7 m), KOMPSAT-3 (0.7 m), Pléiades 1A/1B (0.7 m), SuperView 1-4 (0.5 m), GeoEye-1 (0.46 m), WorldView-1/2 (0.46 m) and WorldView 3 (0.31 m).

The new generation of Earth observation satellites are characterized by an increased acquisition capacity and the possibility of collecting multiple images of the same area from different viewing angles during a single pass [3,4]. This multi-view aspect is essential for extracting 3D information. In recent years, the potential of tri-stereo acquisition from high-resolution satellite images for surface modelling has become an interesting research topic that has been addressed in various publications. The capacity of the Pléiades system in performing 3D mapping was analyzed by Bernard et al. [5], where 17 images acquired from different points of view were used. They showed that by means of “triple stereo” configurations reliable digital surface models can be generated in urban areas. From their best image combination, a root mean square error (RMSE) of 0.49 m was obtained at 295 ground control points (GCPs). The radiometric and geometric characteristics of Pléiades imagery with a focus on digital surface modelling are analyzed by Poli et al. [6]. The model derived from a “triple stereo” scene (nadir, forward and backward) showed median values close to zero and an RMSE in the range of 6–7 m, when compared with a reference light detection and ranging (LiDAR) DSM. An accurate 3D map with tri-stereo images can be obtained by optimizing the sensor models with GCPs, leading to accuracies in the range of 0.5 m in planimetry and of 1 m in height as demonstrated in [7].

Much of the previous research using WorldView-3 satellite images focused on their high resolution multi-spectral information, with applications in topographic mapping, land planning, land use, land cover classification, feature extraction, change detection, and so on [8,9,10,11]. The 3D potential of WorldView-3 data is assessed by Hu et al. [12], where the reconstructed DEM shows height differences of less than 0.5 m for 82.7% of 7256 ground LiDAR checkpoints located along road axes. A new algorithm for generating high quality digital surface models is proposed by Rupnik et al. [13], where a dense image matching method is applied for multi-view satellite images from Pléiades and WorldView-3.

The capability of satellite images regarding a rapid evaluation of urban environments is addressed in Abduelmula et al. [14]. They compared 3D data extracted from tri-stereo and dual stereo satellite images combined with pixel-based matching and Wallis filter to improve the accuracy of 3D models, especially in urban areas. The result showed that 3D models achieved by Pleiades tri-stereo outperformed the result obtained from a GeoEye pair, in terms of both accuracy and detail. This could mean that tri-stereo images can be successfully used for urban change analyses. The potential of VHR optical sensors for 3D city model generation has been addressed in [15,16,17], showing promising results for automatic building extraction when compared to a LiDAR elevation model, although highlighting some difficulties in the case of small individual house reconstruction. A quantitative and qualitative evaluation of 3D building models from different data sources was presented in [18], where a DSM at 1 m resolution derived from a GeoEye-1 stereo-pair, a DSM from an aerial block at 50 cm GSD, and a LiDAR-based DSM at 1 m resolution were used. Their results show that the percentage of correctly reconstructed models is very similar for airborne and LiDAR data (59% and 67%, respectively), while for GeoEye data it is lower (only 41%). The real dimensions of the 17 buildings surveyed were used as ground truth-reference for the 3D building model’s quality assessment, obtaining a mean value for the residual heights of 1.94 m for the photogrammetric DSM. In [19] the authors analyze and validate the potential of high-resolution DSMs produced from stereo and tri-stereo Pléiades-1B satellite imagery acquired over the Athens Metropolitan Area. From their tests, the tri-stereo model shows the best performance in height accuracy, with an RMSE of 1.17 m when compared with elevations measured by a differential global positioning system.

The advantages of using tri-stereo instead of stereo image pairs are described by Piermattei et al. [20], where the nadir image increases the DSM completeness, reducing the occlusions usually caused by larger convergence angles on the ground. Additionally, they investigate in detail the influence of the acquisition geometry (viewing and incidence angles) of VHR imagery on DSM accuracy.

The cited literature concentrates on the accuracy assessments either on open areas or on (large) buildings within city areas, but not on smaller objects like cars.

The standard method of DSM generation from stereo-pairs or triples is dense image matching using global or semi-global optimization. Because of the smoothness constraint of dense image matching [21], the heights of small individual objects may not be reconstructed. Hence, the corresponding 3D points will not have higher elevations compared to their surroundings. Based on this hypothesis, we investigated the capability of dense image matching when evaluating the height of small individual, i.e., detached, objects. While previous studies addressed the general 3D capabilities of VHR sensors, our investigation concentrates on small, isolated object detectability by height estimation. To the best of our knowledge, our study is the first to analyze and quantify the capability of the tri-stereo Pléiades and WorldView-3 reconstructed DSMs for single object detection by height estimation, with focus on vehicles, buildings, and trees. The object’s height compared with its surrounding terrain (in the following simply referred to as height) was investigated with respect to the following parameters: building length and width, vehicle length and width, and tree crown diameter. We investigate DSMs from different sensors with very small GSD, Pléiades and WorldView-3, but the focus is not a comparison of the two satellite systems. Specifically, our research investigation’s purpose is to answer the following questions: (1) which geometric signature and minimum size must individual objects have to be detected in the DSM-based on their reconstructed heights; and (2) what are the influences of different acquisition times, geometries, and GSDs on dense image matching quality for single objects. In the following, we first describe the tri-stereo satellite imagery used together with the study site (Section 2), followed by image block orientation (in Section 3.1) using Rational Polynomial Coefficients (RPCs) and a set of GCPs. Once the orientation is completed, the 3D reconstruction follows. The 3D coordinates of the specific points corresponding to buildings, vehicles and trees are monoscopically measured in all three images: forward, nadir, and backward. The elevations obtained were used for computing the reference individual object’s height, by subtracting the correspondent elevations from a LiDAR digital terrain model (DTM) (in Section 3.2). Subsequently, the accuracy of image orientation and the procedure of dense image matching are detailed in Section 4.1 and Section 4.2. After that, individual objects are grouped into different classes depending on their corresponding sizes. Their automatically-reconstructed heights are then compared with reference values (in Section 4.3 and Section 4.4). Finally, the major findings of the current work are summarized in Section 5.

## 2. Study Area and Data Analysis

**Figure 1:**
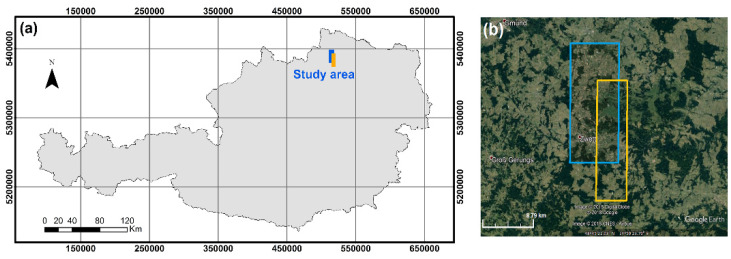
Study area: (**a**) overview map of Austria with location of the study area (coordinates in UTM zone 33N); (**b**) satellite imagery–blue: Pléiades tri-stereo pair and orange: WorldView-3 tri-stereo pair.

The study area is located in Lower Austria, the north-eastern state of the country (48°30′30″ N; 15°08′34″ E; WGS84). With elevations ranging from 537 to 846 m above sea level, the region contains different land cover types such as: urban, suburban, rural, arable, grassland, and forested areas. Analysis was conducted based on tri-stereo satellite images acquired with both Pléiades-1B and WorldView-3 optical satellite sensors. Each triplet consists of images that were collected during the same pass from different sensor-viewing angles (along-track stereo): forward, close to nadir, and backward. The location of the study area acquired with the two sensors, with an overlapping surface of 44.5 km^2^, is shown in Figure 1.

For the current analyses, the reference data contains 43 GCPs measured by means of real time kinematic (RTK) GPS with an accuracy of approximately 1 cm. A DTM generated from a LiDAR flight campaign conducted in December 2015 is available, too. The raster DTM is in UTM 33 North map projection, datum WGS 84 with a grid spacing of 1 m. Its height accuracy was checked against the RTK GCPs yielding a *σ_Z_* of 0.12 m. We used a digital orthophoto from 2017 at 0.20 m resolution for defining the positions of check points (CP). The planimetric accuracy of the digital orthophoto was verified by computing the differences between the RTK point coordinates and their corresponding position on the orthophoto. The result showed that no shifts larger than one pixel were observed.

Each satellite image was delivered with RPCs that allow the transformation between object and image space [22]. Table 1 summarizes the main acquisition parameters like along, across and overall incidence angles, together with corresponding times and covered areas. Additionally, using the equations found in [23] we computed the stereo intersection angles (also called convergence angles in [24]) between each scene and their base-to-height (B/H) ratios. For WorldView-3 satellite imagery the resulting values for the B/H ratios were twice as large as those of the Pléiades imagery.

The tri-stereo Pléiades images were provided at sensor processing level, corrected only from on-board distortions such as viewing directions and high frequency attitude variations [25]. For all three images, we performed an optical radiometric calibration using the open source software Orfeo Tool Box [26]. The pixel values were calibrated by the influence of the following parameters: sensor gain, spectral response, solar illumination, optical thickness of the atmosphere, atmospheric pressure, water vapor, and ozone amount, as well as the composition and amount of aerosol gasses.

In contrast, the WorldView-3 images were delivered as tri-stereo with relative radiometrically-corrected image pixels. The relative radiometric calibration included a dark offset subtraction and a non-uniformity correction (e.g., detector-to-detector relative gain), which is performed on the raw data during the early stages of product generation. We computed the absolute radiometric calibration for each WorldView-3 image. This was done in two steps: the conversion from image pixels to top-of-atmosphere spectral radiance; and the conversion from top-of-atmosphere spectral radiance to top-of-atmosphere reflectance. The calculations were performed independently for each band, using the equations found in the technical sensor description [27]. Usually, the optical radiometric calibration step is necessary before making any physical interpretation of the pixel values. In particular, this processing is mandatory before performing any comparison of pixel spectrum between several images from the same sensor. In our case, this pre-processing step could be omitted, since it did not change the geometric quality of the images.

**Figure 2:**
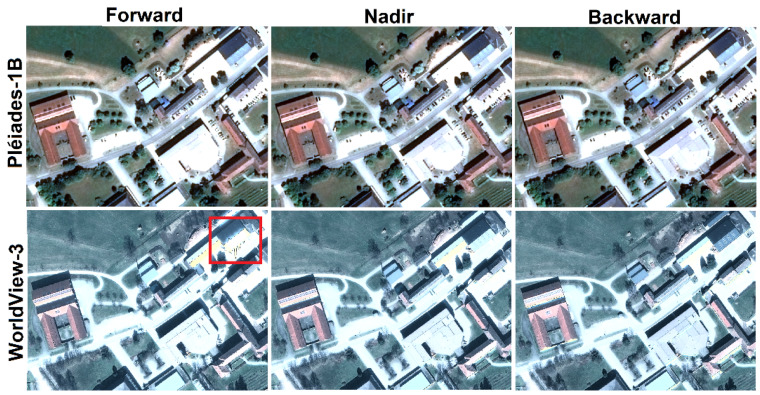
Detail of Pléiades (first row) and WorldView-3 (second row) tri-stereo satellite images on the same built-up area, acquired with forward-, nadir- and backward-looking.

An important characteristic of both satellites—Pléiades-1B and WorldView-3—is the fast rotation to enable recording three images within the same orbit in less than 1 min. Therefore, the images have the same illumination conditions and shadow changes are not significant. With radiometric resolutions of 16 bit/pixel, the images provide a higher dynamic range than the traditional 8- or 12-bit/pixel images. From a visual inspection, some radiometric effects were observed in the WorldView-3 forward image (Figure 2). Here, the reflective roof surface, in combination with the imaging incidence angle and sun elevation, caused saturation and spilling effects. In contrast, no radiometric artefacts were observed in the Pléiades satellite images.

### 2.1. Pléiades-1B Triplet

The Pléiades satellite system is composed of two identical satellites, Pléiades 1A/1B, which were launched by the French space agency (CNES) in December 2011 and December 2012, respectively. Both satellites are flying on the same sun-synchronous low-Earth orbit at an altitude of 694 km with a phase of 18° and an orbital period of 98.8 min. The sensors are able to collect both panchromatic and multispectral images [3].

The Pléiades-1B tri-stereo images used in the current investigations were acquired in the late morning of 13 June 2017 within 23 s. Within this time, the satellite travelled a total distance (Base) of 167.87 km, leading to an intersection angle of rays on the ground of 12°. The B/H ratios are of 0.10, 0.11, and 0.21 for forward-nadir, nadir-backward and forward-backward image combinations. The pansharpened images composing the triplet are available in 16 bit, each of them with four spectral bands, i.e., red, green, blue, and near-infrared. Depending on the viewing angle, the mean values for the GSD vary between 0.70 and 0.71 m.

### 2.2. WorldView-3 Triplet

The WorldView-3 Digital Globe’s very high-resolution optical sensor was launched in August 2014. Operating at an altitude of 617 km with an orbital period of 97 min, the sensor provides 0.31 m panchromatic resolution.

According to the metadata, the tri-stereo WorldView-3 images for our study were acquired in spring 2018, on 8 April, within 37 s. The corresponding base has a value of 279.69 km and the intersection angle of rays on the ground is 23°. Even though both sensor platforms fly at approximately the same speed (~7.5 km/s), the WorldView-3 B/H ratios have higher values, because of the lower altitude and increased acquisition time, compared to the Pléiades triplet. Hence, the B/H ratio is 0.20 for both forward-nadir and nadir-backward images, whereas for forward-backward it is 0.40. Each image is pan-sharpened, with four spectral bands, i.e., red, green, blue, and near-infrared, with 16-bit depth and zero cloud coverage. Depending on the viewing direction, the mean GSD values vary from 0.31 m to 0.32 m. The images were delivered as eight, two, and two tiles for close-to-nadir, forward, and backward, respectively. As a pre-processing step, the tiles for each image were mosaicked accordingly. For both image triplets, Pléiades and WorldView-3, auxiliary data including the third-order rational function model (RFM) coefficients are provided as separate files.

**Figure 3:**
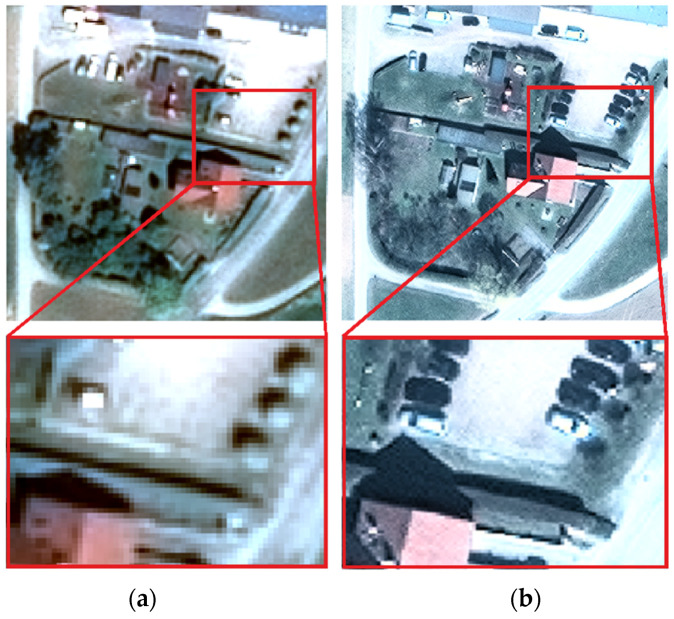
Comparative view of the same area from Pléiades (**a**) and World View-3 images (**b**) with a magnified detail.

Figure 3 shows a visual comparison between the pan-sharpened (near) nadir images acquired with Pléiades (left) and WorldView-3 (right) over a small area, in order to highlight the effects of the two different resolutions of 0.70 m and 0.31 m. The same objects can be distinguished in both images: streets, buildings, cars, and trees. The higher resolution of WorldView-3 provides a more detailed image with clear visible cars, tree branches, and building roof edges.

## 3. Data Processing and Analyses

### 3.1. Image Orientation

The prerequisite for a successful photogrammetric 3D reconstruction is that both interior and exterior orientations are correctly known. The physical sensor model based on the collinearity condition, describing the geometric relation between image points and their corresponding ground points, yields high accuracies (typically a fraction of one pixel), but is complex, and varies depending on different sensors types. Moreover, information about the camera model, ephemerides, and satellite attitude may not be available to users, since they are kept confidential by some commercial satellite image providers [28]. Usually, in practice, the RFM containing eighty RPCs is used for replacing the rigorous physical model of a given sensor [29]. In our research work, we have used the RFM for the photogrammetric mapping.

The workflow for DEM extraction from tri-stereo images begins with image block triangulation and geo-referencing, based on provided RPCs and available GCPs. The main steps for the satellite triangulation are:

1. Image point measurement of GCPs. The number of GCPs is different for the two image sets because of the different data extent and visibility in the scenes. Therefore, 43 GCPs and 22 GCPs were manually measured in each Pléiades and WorldView-3 tri-stereo pair using the multi-aerial viewer, which allows a simultaneous display of the images. This step is performed in order to stabilize the image block and to achieve higher accuracies by improving the given RPCs’ values.
2. Tie points (TPs) extraction and RPC refinement. The orientation of the satellite imagery includes the automatic TPs extraction as well as an automatic and robust block adjustment. During the adjustment, a maximum number of six parameters (affine transformation in image space) can be computed: two offsets, two drift values, and two shear values (for each image). Depending on their significance, only a subset of these corrections could be computed by the software: two shifts (on X and Y) and a scale on Y. TPs were automatically extracted by applying Feature Based Matching using the Förstner operator and refining them with Least Squares Matching [30]. TPs with residuals (in image space) larger than one pixel are considered mistakes (gross errors) and rejected. The RPCs are refined through a subsequent adjustment procedure where the differences between image- and backprojected- (with the RFM) coordinates of the GCPs and TPs are minimized.
3. Geo-positioning accuracy analysis. To evaluate the accuracy of the georeferenced images, 50 CPs were used. They were manually acquired from the available orthophoto at 0.2 m GSD and their heights were extracted at the same locations from the LiDAR DTM (1 m resolution). For CP selection, stable details on the ground such as road marks (e.g., pedestrian crossing lines), road surface changes, corners of paved areas and corners of parking lots were selected. Considering the horizontal accuracy of the orthophoto (0.10 m) and the vertical accuracy of the DTM (0.12 m), these points are less accurate than the RTK point measurements.

For the current work, the entire photogrammetric workflow was implemented in the Inpho 8.0 software from Trimble [31], designed to perform precise image block triangulation through bundle block adjustment and 3D point cloud reconstruction using dense image matching techniques for push-broom cameras. The same processing line was followed for Pléiades and WorldView-3 tri-stereo images.

### 3.2. Manual Reference Measurements

After the image orientation was completed, the manual measurement of the 3D points was performed. For each object, the highest point was selected, such as points on a building’s ridge, on a car’s roof and tree crown centre (approximation of the tree top). For the 3D restitution, we manually measured the points monoscopically in all three oriented images (forward, nadir and backward), in a multi-aerial view mode. The mean-square error of the Z object coordinate is given by the following formula [32]:

(1) $$\sigma_{Z}=\sqrt{2}\cdot m_{B}\cdot \sigma_{B}\cdot \frac{Z}{B},$$

with *σ_Z_* the object elevation accuracy, *σ_B_* the accuracy of image measurement (1/3rd of a pixel), *Z* the altitude of the satellite orbit, *B* the base and *m_B_* the satellite image-scale number given by the *Z/c* ratio, where *c* is the focal length of the optical system. Due to the large heights, we considered the special case of parallelism between object and image plane; hence a single scale number for the whole satellite image was used.

Taking into account these parameters, the estimated accuracy of the *Z* object coordinates is 1.36 m and 0.31 m for the Pléiades and WorldView-3 images, respectively. These results suggest a minimum object height of 1 m as a reference height that guarantees a reasonable analysis. Since the smallest investigated cars have around 1.5 m height and buildings and single trees usually have more than 2 m height, the estimated elevation accuracy does not significantly influence our investigations. 

In a final step, the reference object heights are computed by using the measured *Z*-coordinates and the elevations extracted from the LiDAR DTM (with 1 m resolution and *σ_Z_* of 0.12 m) at each point’s position. Assuming that the measured and extracted *Z* values have random and uncorrelated errors, according to the law of error propagation, the uncertainty associated with the manual measurements for reference object height computation is determined by the quadrature of input elevation uncertainties.

The geometric parameters, i.e., vehicle length/width, tree crown diameter and building length/width, were directly measured on the rectified images in a geographic information system (GIS) environment.

Three different classes of objects were separately investigated:

(a) Vehicles are classified into four groups depending on their length: (1) passenger and family car type, smaller than 5 m; (2) vans having lengths between 5 and 7.5 m; (3) trucks with lengths between 7.5 and 10 m; and (4) articulated lorries, trailers and multi-trailers usually having more than 10 m. The lengths, widths and the corresponding elevations at car’s roofs of 50 vehicles are investigated. The computed mean reference heights are 1.5, 2.5, 3.7, and 4 m for family cars, vans, trucks, and articulated lorries, respectively. Related to average object height, the associated uncertainty of the manual height measurement varies between 8% for lorries and 22% for family cars for WorldView-3 and between 34% and 68% for Pléiades.
(b) Trees are typically classified into two categories: coniferous and deciduous. Coniferous trees are cone-shaped trees, represented mainly by spruce in our case. The second category is the broad-leaved trees with leaves that fall off on a seasonal basis, mostly represented by beech and oak. We needed to perform this classification due to the different acquisition times: one in June (leaf-on conditions) and the other one in April (leaf-off conditions). The diameter and elevations were measured for 100 trees (50 trees for each category: deciduous and coniferous). Depending on crown diameters, they were grouped into seven categories, beginning with trees with a crown smaller than 2.5 m, and ending at large trees with 10 m diameter. The computed mean reference heights for the seven coniferous tree classes are: 5.5, 7.8, 11.0, 14.6, 17.2, 23.4, and 28.7 m, with uncertainties between 1% and 6% from object height for WorldView-3 and between 5% and 24% for Pléiades. The mean reference heights for the deciduous tree classes are: 3.1, 5.4, 8.0, 12.6, 15.4, 16.2, and 18.5m, with uncertainties between 2% and 10% from object height for WorldView-3 and between 7% and 44% for Pléiades.
(c) For buildings, two geometrical parameters are taken into account: length and width. According to their size, built-up structures are grouped into several classes starting with very small (5 m in length and width) to large (50 m length and 25 m width). Therefore, lengths, widths, and roof ridge elevations were measured for 100 buildings in both Pléiades and WorldView-3 images. The mean reference height values varied from 2 m (small built-up structures) to 10–12 m (large industrial buildings), with associated uncertainties from 2% to 16% from object height for WorldView-3 and between 11% and 68% for Pléiades.

While identical trees and buildings were investigated in both Pléiades and WorldView-3 images, this was not possible for vehicles, since they are moving objects. Therefore, (parked) vehicles were randomly selected within the entire scene also using the non-overlapping parts.

### 3.3. Satellite Image Matching and 3D Reconstruction

Image matching algorithms were used to identify homologous objects or pixels within the oriented images. These algorithms can be divided into area-based (e.g., [33]), feature-based (e.g., [34]), and pixel-based matching (e.g., [35]). Alternatively, image-matching approaches are often classified into sparse and dense matching or into local and global matching methods. The automatic image matching and DSM reconstruction processes were individually performed for each tri-stereo-scene (Pléiades and WorldView-3) by using the specialized module of the Inpho software, called Match-T DSM. The DSM derivation was based on three matching strategies [30]: (a) least squares matching (LSM); (b) feature-based matching (FBM) [36]; and (c) cost-based matching (CBM). Like in most image matching procedures, where image pyramids were used to reduce computation time [37], in our case, the iterative processing chain contains ten pyramid levels. On each pyramid level, three processes were performed: the matching of homologous image points, 3D intersection in object space and DEM modelling. For the first seven pyramids, FBM was used, and the last three image pyramid levels were processed with CBM. CBM is a pixel-by-pixel matching technique similar to the semi-global matching algorithm [35,38]. The CBM strategy within the Match-T DSM module uses a search-path in a so-called 3D-cost-cube for finding the corresponding pixels in images. The cost functions (e.g., correlation coefficient) are used to find the minimum cost path, and each direction represents an *X*–*Y* movement in the image to match. Finding the pixel with the lowest cost generates a lowest-cost 3D model–surface model [30]. For each image pixel, the 3D object point coordinates were calculated by applying forward intersections, finally resulting in dense photogrammetric point clouds for the entire study area.

In the last step, high resolution DSMs were generated by using the robust moving planes interpolation method. For each grid node all points within a circular neighborhood are used to robustly estimate a best fitting tilted plane (minimizing the vertical distances); points classified as outliers are not considered in the plane fitting procedure. This step was performed with the scientific software OPALS (Orientation and Processing of Airborne Laser Scanning data) [39].

## 4. Results and Discussion

### 4.1. Block Triangulation

During the satellite triangulation of the tri-stereo scene, TPs were automatically extracted in each Pléiades image. They were homogenously distributed within the forward, nadir and backward images, with standard deviations of the adjusted coordinates for elevations ranging from 1.49 m (2.1 GSD) to 3.49 m (5.0 GSD) (Table 2). The standard deviations of the TP elevation obtained for theWorldView-3 scenes are clearly better relative to the GSD, with a maximum value of 0.74 m (2.5 GSD). The standard deviation of the satellite image block-triangulation was 0.54 pixels for Pléiades images and 0.46 pixels for WorldView-3.

We evaluate the accuracy of the estimated orientation with respect to the measured GCPs and CPs. The root mean square error values of their coordinates in units of meters and pixels are shown in Table 3. For both sensors the RMSE values of the GCPs for latitude, longitude and elevation are at sub-pixel level, showing that the block adjustment provides precise results for subsequent processing. The small planimetric discrepancies suggest that the GCP identification in images is better than 1/3rd of a pixel. The highest discrepancy values for the CP height are 0.85 m (1.21 GSD) for Pléiades and 0.28 m (0.93 GSD) for WorldView-3. The Pléiades elevation accuracy (0.85 m) is not as good as the one in [5], which resulted to 0.49 m, but is more favorable than the results in [7], where 1 m RMSE in elevation were reported. For WorldView-3, the vertical accuracy of 0.28 m fits to the results in [12], which reported an elevation bias of less than 0.5 m for 6001 ground LiDAR CPs. The residual errors for Pléiades images at CPs are comparable with the results obtained by [40] reporting RMSEs of 0.44 m, 0.60 m and 0.50 m at 40 GCPs for latitude, longitude and elevation, respectively.

### 4.2. Dense Image Matching

The dense image matching algorithm was applied individually to the two sets of image triplets available as 4-band pansharpened products. Using four cores of a 3.50 GHz machine with 32 GB RAM, the 3D point clouds were generated in 10 and 33 h for Pléiades and WorldView-3 datasets, respectively. The direct output are dense 3D photogrammetric point clouds in the LAS file format with one point for each image pixel. In the overlapping area, the Pléiades-based point cloud consists of 169 million points, whereas the WorldView-3-based equivalent has 476 million points. Hence, it’s almost three times denser. Overall, the reconstructed point clouds have a regular distribution on ground plane domain, with densities of 4 points/m^2^ and of 12 points/m^2^ for Pléiades and WorldView-3, respectively. A few small regions were not reconstructed due to occlusions (e.g., large elevation difference between buildings/trees and surrounding ground). Nevertheless, these holes were filled using interpolation algorithms in the following step.

Based on a neighborhood search radius of 1 m and a maximum number of 20 nearest neighbors, we interpolated a regular grid structure using robust moving planes interpolation. For each dataset, a digital surface model was generated using the same parameters. Since we wanted to use the direct output of the 3D reconstruction without losing any information, the point clouds were not edited or filtered before the interpolation step.

Usually, interpolation strategies tend to smooth the initial elevation values of the 3D point cloud. In order to minimize this effect, we selected a small size of the grid cell (0.5 m × 0.5 m) and a relatively small neighborhood definition for the interpolation. To determine the direct degree of smoothing, a raster containing the maximum elevations in each 0.5 m × 0.5 m cell was computed. Using this as a reference, the 2.5D Pléiades interpolated model showed an RMSE value of 0.079 m, while the WorldView-3 DSM had 0.098 m. The latter was slightly higher, but these values (at sub-decimetre level) still showed that the smoothing effect of the interpolation will not significantly influence further analysis. 

The vertical quality of the photogrammetrically derived DSMs was evaluated against the available elevations of the GCPs and CPs (Table 4). The computed mean values for the CPs—0.07 m (Pléiades) and 0.01 m (WorldView-3) and the standard deviations of 0.30 m (Pléiades) and 0.13 m (WorldView-3)—are comparable with the results obtained by [13], who reported mean values ranging between 0.08 and 0.29 m and standard deviations between 0.22 and 0.53 m.

For Pléiades, we obtained an RMSE in the *Z*-direction of 0.29 m for GCPs and of 0.31 m for CPs. The WorldView-3 DSM showed a higher vertical accuracy, with RMSEs of 0.12 m and 0.13 m for GCPs and CPs, respectively. This is because the vertical accuracy of the DSM is directly influenced by the triplet acquisition geometry, especially by the intersection angle on the ground. As described in [41], the vertical accuracy of the DSMs from VHR satellite imagery is influenced by the acquisition geometry, where a wider angle of convergence (>15°) enhances the height accuracy. In our case, the Pléiades scenes with a narrow convergence angle (12°) show a lower vertical performance than the WorldView-3 scenes, with a larger convergence angle on the ground (23°).

The two high resolution DSMs derived from Pléiades and WorldView-3 tri-stereo scenes within the WGS84-UTM33 reference system were used as source data for the following single object investigations.

### 4.3. Object Height Differences

For a clear height estimation, in our work we considered only single objects, located in free, open and smooth areas, without any other entities in their close surroundings. Assuming that in the free, open areas, the DSM coincide with DTM elevations, we inspected the vertical quality of the photogrammetric derived surface models by computing the vertical offsets from the reference LiDAR DTM (1 m resolution and *σ_Z_* = 0.12 m). The results showed a good correspondence of the DSMs with the reference DTM, featuring a RMSE of 0.32 m for Pléiades and of 0.20 m for WorldView-3. When it comes to individual objects, we sometimes observed a constant offset, but it was always below 0.30 m. Therefore, it did not significantly impact our height investigations.

The reconstructed individual object height was extracted from the interpolated surface models. Heights of buildings, vehicles and trees were computed by subtracting the DTM elevations from the elevations of the highest object points (located on roof ridges, on car roofs, and on treetops) at the same 2D location.

We consider the reference height (*H*) as being the real object height, which was computed by subtracting the DTM elevation from the manual measurements (Section 3.2) at the same position.

*H* = *Z_M_* − *Z_DTM_* (*m*) (2)

*h* = *Z_DSM_* − *Z_DTM_* (*m*), (3)

with *H* the reference object height, *h* the estimated object height, *Z_M_* the manual elevation measurements, *Z_DTM_* the elevation of the ground surface and *Z_DSM_* the elevation of the reconstructed DSM.

In Equations (2) and (3) the *Z_DTM_* elevation values are identical, since for computing the reference and the estimated heights, the same ground elevation at the same 2D position was considered. Based on the defined equations, we obtained a reference height (*H*) and two estimated heights for each individual object (from Pléiades and WorldView-3 DSMs). These values will be employed in further analysis.

### 4.4. Height Estimation of Single Objects

Within this investigation, we wanted to estimate the Pléiades and WorldView-3 DSMs heights at single objects as a function of object type and size. For this purpose, three object types were analyzed: non-moving vehicles, trees, and buildings. The main parameters taken into account were vehicle length/width, tree crown diameter, and building length/width. 

For each single object, the estimated heights were compared with the monoscopic reference measurements based on a ratio measure. The percentage value derived by the following equation describes how much of the reference height was reconstructed:

*p* (%) = *h*/*H* × 100, (4)

with *H* the reference height, *h* the estimated height, and *p* the reconstruction percentage.

#### 4.4.1. Vehicles

**Figure 4:**
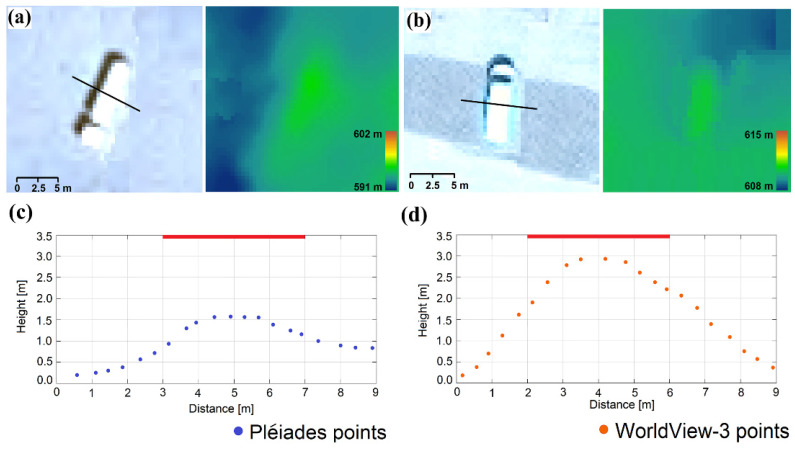
Height estimation for a truck. (**a**) Pléiades satellite image detail with profile location (left) and reconstructed DSM Z color-coded (right); (**b**) WorldView-3 satellite image detail with profile location (left) and reconstructed DSM Z color-coded (right); (**c**) Pléiades-; and (**d**) WV3-based vehicle profiles of 0.5 m width each from the matched point cloud; the red horizontal line is the corresponding measured reference height.

By a visual inspection of the reconstructed Pléiades-based 3D point cloud, it was observable that points corresponding to small vehicles (vehicles with lengths smaller than 5 m) were not elevated against their surroundings. Larger vehicles were reconstructed in height, but not entirely (Figure 4).

Specifically, Figure 4 represents a truck with a length of ~10 m and a real height of 3.5 m that is visible in both Pléiades (a) and WorldView-3 images (b). On the left-hand side are the magnified satellite image details of the vehicle and on the right-hand side are the corresponding reconstructed height color-coded DSM. From the DSMs and the cross profiles (c), it is clearly visible that in contrast to Pléiades, where there is only ~1 m elevation change, the WV3-based profile reconstructs the vehicle height up to 2.9 m, which represents ~83% of the real height.

**Figure 5:**
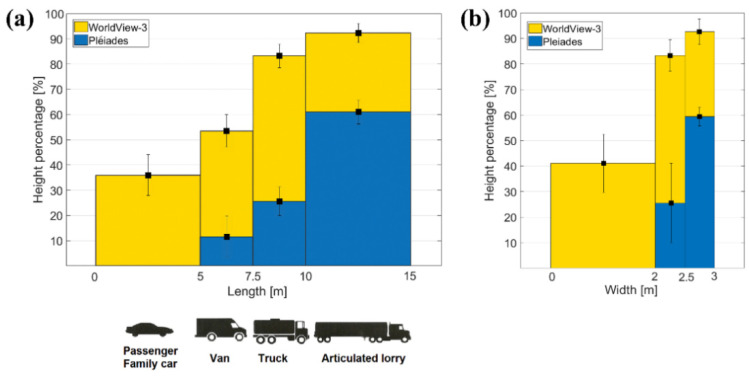
Interval-based percentage and standard deviation for height estimation of vehicles depending on (**a**) lengths and (**b**) widths.

Using Equation (4), the height percentages of 50 measured vehicles were computed for both cases: Pléiades and WorldView-3 sensors. As mentioned in Section 3.2, vehicles were classified into four different groups depending on their lengths, starting with small family cars, of approximately 5 m, followed by vans, trucks, and finally by large articulated lorries with more than 10 m length. For each group, containing between 10 and 13 vehicles, the mean value of the reconstruction height percentage and the standard deviation were determined. They are shown in Figure 5a for both Pléiades (blue) and WorldView-3 (yellow) data. By increasing length, the height percentage also increases, reaching up to 60% (Pléiades) and 92% (WorldView-3). In the case of the Pléiades imagery, small vehicles (<7 pixels length in image) do not show any height information, while for family cars, vans, and trucks the reconstruction height percentage is less than 30%. It exceeds 50% only for large vehicles (lengths >10 m, 14 pixels in the image). On the other hand, in WorldView-3 DSMs, vehicles have a good height reconstruction (reaching over 90% for lengths >10 m, 33 pixels in image). An exception are family cars (~16 pixels length), which have a percentage of less than 40%. The standard deviations of the estimated heights are notably smaller than the overall trend and they become smaller with increasing vehicle length.

A similar analysis was conducted by using vehicle width as the main parameter for vehicle grouping (Figure 5b). Because small family cars and vans have similar widths (~2 m), they form a single class, followed by trucks (2 to 2.5 m widths), and articulated lorries (2.5 to 3 m widths). Based on the WorldView-3 DSMs, vehicles with up to 2 m widths have ~41% of their real height reconstructed. This value is higher when compared with the percentage of the first length-class (37%) because it contains both family cars and vans, leading to an increased mean height percentage and standard deviation. The mean height percentages reach 59% (Pléiades) and 92% (WorldView-3) for the very large vehicles. The increased values of standard deviations suggest a higher variation of the reconstruction height percentages within the width-classes considered. Therefore, the length parameter is more suitable for the description of the vehicle’s height estimation.

#### 4.4.2. Trees

**Figure 6:**
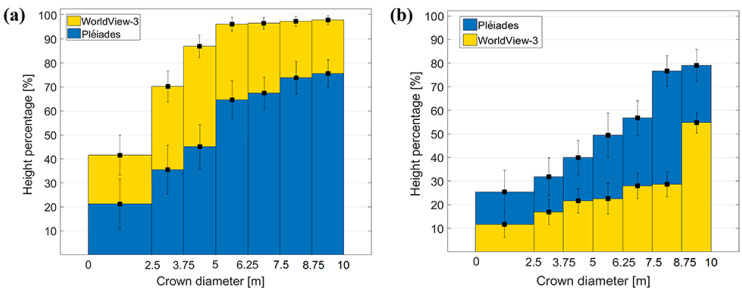
Interval-based percentage and standard deviation for height estimation of single trees (**a**) coniferous trees and (**b**) deciduous trees.

Trees were investigated in two separate classes: deciduous trees, located especially on roadsides, parks and cities, and coniferous trees, found in forested areas and forest patches. Only single, isolated trees, not close to buildings or any other objects, were taken into consideration. In this case, the visible crown diameter was used as the geometric parameter. One hundred trees were investigated, and the same formula for height percentage computation, Equation (4), was applied. Based on diameter, they were grouped into seven different categories, each containing approximately 12 trees. The mean reconstruction percentages gradually increase with crown diameter and the standard deviations decrease (Figure 6). For WorldView-3, the heights of coniferous trees with crown diameters <2.5 m (8 pixels in image space) are reconstructed less than 50%, whereas trees with crown diameters larger than 5 m (16 pixels in image space) are over 95%. Generally, heights of coniferous trees are better estimated from WorldView-3 than from the Pléiades DSMs, where trees with diameters >7.5 m (11 pixels) barely reach 75% height reconstruction. For deciduous trees (Figure 6b) it can be observed that the situation is reversed: they get better height information from Pléiades DSM, than from WorldView-3 DSM. This is mainly because of the different acquisition times, June for Pléiades and April for WorldView-3, indicating leaf-on and leaf-off conditions which clearly influence the appearance and texture of the trees in the images. For the deciduous trees, only the stems and branches are visible in the WorldView-3 images, resulting in a poor dense image matching quality. For trees with a crown diameter larger than 9 m, heights of just over 50% of the tree values are reconstructed.

When analyzing the two tree types only from the Pléiades images (Figure 6), we could see that there is a slightly better height reconstruction for the very small and large deciduous trees, compared with the coniferous. The small deciduous trees in the first group (with crown diameters smaller than 2.5 m) have 26% of their real height reconstructed, whereas the small coniferous trees in the first group have only 21%. The same is true for the last group, where large deciduous trees (with crown diameters bigger than 8.75 m) have percentages close to 80%, whereas for coniferous they reach only 75%. On the other hand, for the coniferous trees of medium sizes (with crown diameters between 2.5 and 7.5 m) we obtained better height reconstruction percentages than for the deciduous.

**Figure 7:**
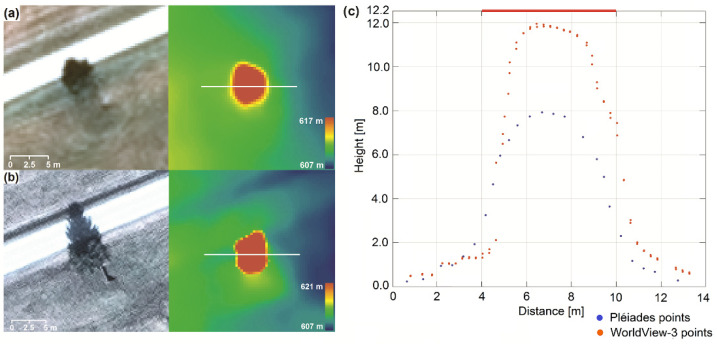
Height estimation of a coniferous tree with 7 m crown diameter: (**a**) Pléiades satellite image detail (left), corresponding height-colored DSM and profile location (right); (**b**) WorldView-3 satellite image detail (left), height-colored DSM with profile location (right); and (**c**) tree profile of 0.5 m width from the matched point clouds; the red horizontal line is the measured reference height.

**Figure 8:**
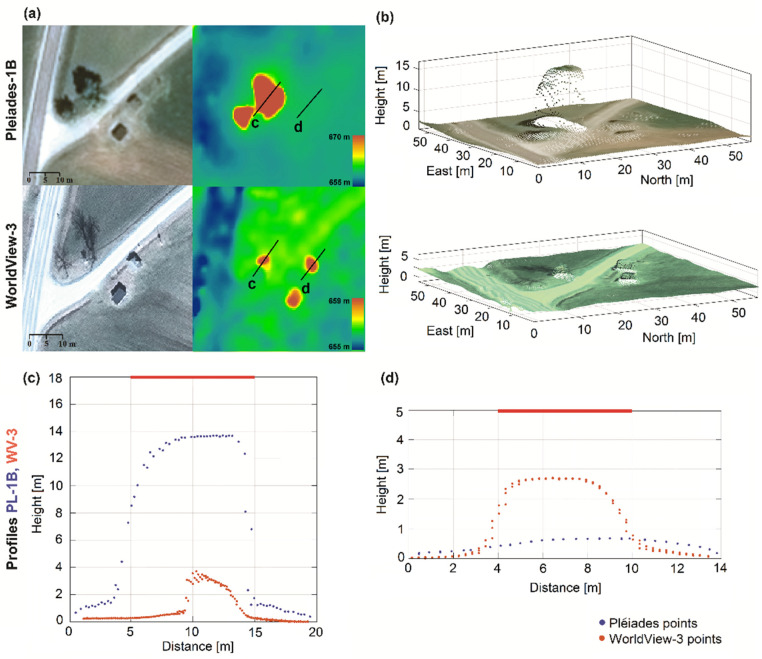
Height estimation of a deciduous tree and a small building: (**a**) Pléiades (first row) and WorldView-3 (second row) views with corresponding height–color coded DSMs; (**b**) RGB colored 3D point clouds, 1 m width height profiles from the matched point clouds; (**c**) for a deciduous tree; (**d**) for a small building; the red horizontal lines are the corresponding measured reference heights.

Two examples of height estimation of a coniferous (Figure 7) and a deciduous tree (Figure 8) are shown. They were comparatively analyzed for Pléiades and WorldView-3 satellite images based on both DSM and point cloud. For single coniferous trees with a 7 m crown diameter (Figure 7) we achieved different results based on the input data. From the true height of 12.2 m, the estimation resulted in approximately 12 m for WorldView-3 and only 8 m for Pléiades, which represents 98% and 65% of the real height.

A small area of the DSMs and the 3D point clouds generated from the Pléiades and WorldView-3 satellite imagery are shown in Figure 8. The largest deciduous tree with leaves (Figure 8a first row) has a reasonable appearance in the DSM and an estimated height of 14 m. Meanwhile, the same leafless tree in the WorldView-3 image (Figure 8a second row) has a height of only approximately 4 m. A visual analysis of height differences for this deciduous tree and a small building (next to the tree) is shown in two profiles (c) and (d) in Figure 8.

#### 4.4.3. Buildings

The third category of objects investigated were single buildings, usually in suburban and rural areas. They usually had regular geometric shapes, such as rectangles or squares, which facilitated the visual identification of the corresponding dimensions in the rectified images. The building length/width were measured in the rectified images, based on their roof top view. For buildings with a T- or L-shaped plan, we considered the length as the longest dimension between extreme roof edges, whereas the width as the distance across the roof from one side to the other. One hundred buildings were chosen to cover a wide range of dimensions from 1 to 50 m length (large industrial buildings) and widths from 1 to 25 m. In the example shown in Figure 8b, the two small buildings are elevated against their surroundings in the WorldView-3 data, which is not the case for the Pléiades data. In addition, the profile (d) with two overlaid point clouds corresponding to a 3.6 by 2.5 m building clearly reveals the higher potential of WorldView-3 sensor for height reconstruction, as opposed to Pléiades. Even if the building edges appear very smooth, the corresponding main height is still reconstructed.

**Figure 9:**
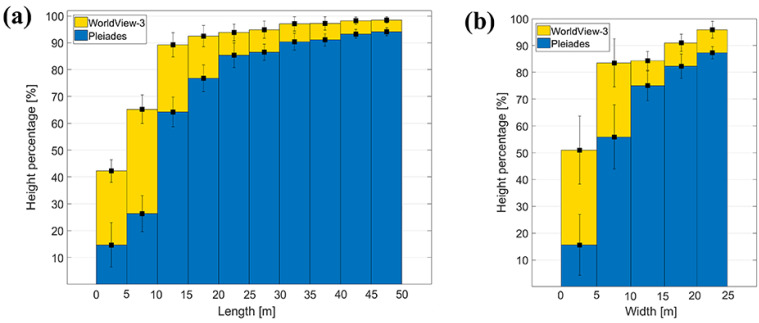
Interval-based percentage and standard deviation for height estimation of buildings depending on (**a**) lengths; (**b**) widths.

Again, Equation (4) was applied for investigating the potential of Pléiades and WorldView-3 DSMs for building’s height estimation with respect to their lengths and widths (Figure 9). Based on their length, we defined ten different categories, each containing approximately ten buildings (Figure 9a). Like in the previous analyses for vehicles and trees, the computed mean height percentages for buildings show a similar trend, values gradually increasing with building length. In case of the Pléiades imagery, buildings with lengths smaller than 10 m (14 pixels in image space) have a height reconstruction percentage of less than 30%, whereas buildings with lengths greater than 5 m have percentages beyond 50%. For both Pléiades and WorldView-3, buildings with lengths >43 pixels have over 90% height reconstruction.

Due to the lower variation in building-width, we considered only five intervals, starting from 0–5 m to 20–25 m (Figure 9b). Compared with height-percentage analysis based on building length, for the width parameter the situation was different. When buildings were grouped based on their corresponding widths, we had an increased number of items within each interval (only 5 width-intervals). This led to a higher variation of height estimation percentages, and hence to higher values for the standard deviations within each interval. When analyzing the reconstruction height percentages based on the WorldView-3 images for both 0–5 m and 5–10 m length/width intervals, we could see higher mean values for the reconstruction percentages computed based on building widths. Nevertheless, the corresponding standard deviations are larger in contrast to those computed on the length basis. The standard deviations for the length-intervals (Figure 9a) become smaller with increasing building length, showing a clearer trend; therefore, the length parameter gives a better estimation when analyzing the height reconstruction of buildings.

**Figure 10:**
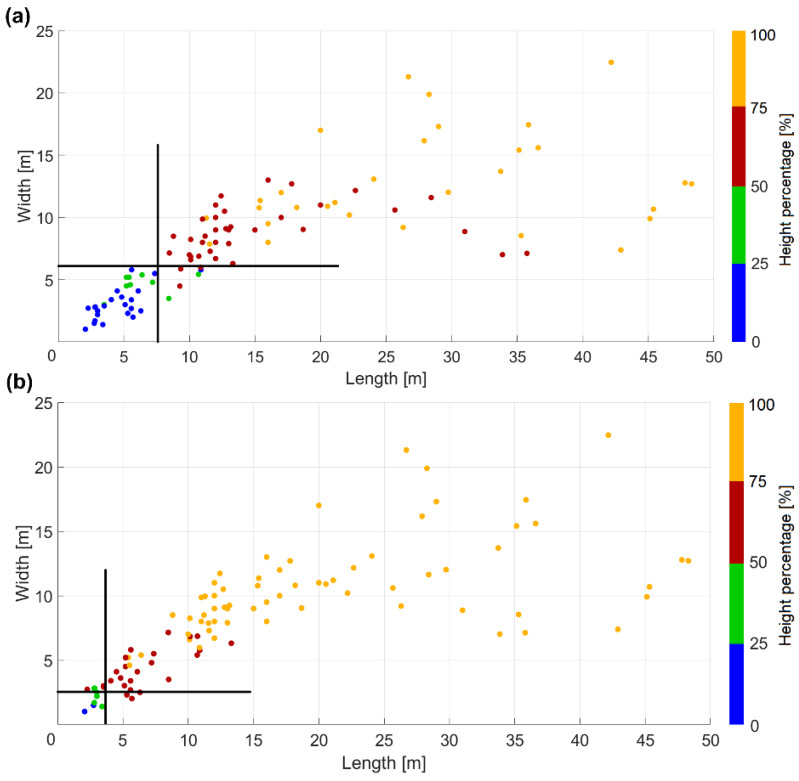
Height estimation of buildings: (**a**) for Pléiades; (**b**) for WorldView-3.

The two scatter plots (Figure 10) show the relationships between building length and width and the reconstruction height percentage. As expected, height accuracy is better for WorldView-3 images than for the Pléiades data. The delineation lines separate the buildings with higher and lower reconstruction height percentage (p) than 50%. If for Pléiades data a 2D minimum building size of 7.5 m length by 6 m width is needed in order to get *p* > 50%, for WorldView-3 data only 4 m × 2.5 m is needed.

The mean value for the residual heights corresponding to reconstructed buildings with *p* > 50% (of 2.02 m for Pléiades with 0.7 m GSD) is comparable with the residuals of 1.94 m obtained by [18] when using a DSM from a GeoEye stereo pair (0.5 m GSD).

## 5. Conclusions

This study addresses the potential and limitations of both Pléiades and WorldView-3 tri-stereo reconstructed DSMs for height estimation of single objects. Satellite image orientation and dense image matching were performed. The tri-stereo images of each sensor were acquired in less than one minute, resulting in similar radiometric characteristics, a fact that is beneficial for any image matching algorithm. For obtaining higher accuracies (at sub-pixel level in image space and sub-meter level in object space) the direct sensor orientation available as RPCs is improved by GCPs through a bias-compensated RPC block adjustment. Evaluated at 50 CPs in open areas, elevation accuracies of 0.85 m and 0.28 m (RMSE) were obtained for Pléiades and WorldView-3, respectively.

Next, photogrammetric DSMs were generated from the Pléiades and WorldView-3 tri-stereo imagery using a dense image matching algorithm, forward intersections, and interpolation. The estimation of a single object’s height depends on the accuracy of satellite image orientation, but also on the elevation accuracy of the derived DSMs and on the LiDAR reference DTM. We found that the vertical accuracy of the reference DTM w.r.t the RTK GCPs is 0.12 m, and the accuracies of the reconstructed photogrammetric models with respect to the DTM are 0.32 m for Pléiades and 0.20 m for WorldView-3 in open, smooth areas. The vertical accuracy of the DSMs from VHR satellite imagery is influenced by the acquisition geometry. The Pléiades scenes having a narrow convergence angle of 12° show a lower vertical performance compared to WorldView-3 scenes, which have a 23° convergence angle on the ground. In addition, the smoothness effect of the robust moving planes interpolation for deriving the DSMs is at sub-decimetre level. Under these conditions, the resulting height accuracy is reasonable and does not significantly influence our investigations, since the single objects measured are usually higher than 1.5 m. Based on the different GSDs and B/H ratios of the two satellite systems, it can be said that *σ_o_* of block adjustment, RMSE of check point elevation error and height reconstruction success for individual objects are similar, with deviations of up to 20%.

In order to investigate the potential of Pléiades and WorldView-3 tri-stereo DSMs for single object height estimation, 50 vehicles, 100 trees, and 100 buildings were analyzed. Vehicle length/width, tree crown diameter and building length/width were identified as the main geometric parameters for classifying single objects into different groups. The results clearly show a gradual increase of the estimated heights with object dimensions. Starting from very small objects (whose heights are not reconstructed) the values for the height reconstruction percentage reached almost 100% for very large single objects (e.g., buildings with lengths >35 m in the case of WorldView-3 DSM). Hence, individual objects’ reconstructed heights strongly depend on object type, size, and the sensor being used. As an example, if we consider the *p* = 50% threshold for an appropriate height reconstruction, then we obtain the following minimum size values for the single objects: 10 m (Pléiades) and 5 m (WorldView-3) for vehicle lengths; 5 m (Pléiades) and 2.5 m (WorldView-3) for tree crown diameters and 7.5 m × 6 m (Pléiades) and 3.5 m × 2.5 m (WorldView-3) for building length/width. The ratio of these dimensions fits well to the GSD ratio of 70 cm to 31 cm.

Generally, for all single objects except deciduous trees, the results achieved with WorldView-3 data were better than those achieved by using the Pléiades data. Hence, the ability of WorldView-3-derived DSM to perform height estimation of single objects is higher compared with Pléiades DSM. This is mainly because of the very high resolution (0.31 m) that leads (through photogrammetric processing) to very dense 3D point clouds, describing the real ground surface and the objects on it more accurately. The acquisition times also play a significant role in the photogrammetric procedure. For stable objects such as buildings, the different periods of acquisition do not affect the 3D reconstruction, but for deciduous trees, the automatic reconstructed heights are highly underestimated in the leaf-off season. Their leafless appearance in the images brings difficulties to the image matching process when finding correct correspondences for tree branches. From this observation, we can conclude that the image appearance and texture of trees (and other single objects) are also very important for the 3D reconstruction and height estimation. For all objects investigated, the resulting histograms (Figure 5, Figure 6 and Figure 9) show interesting trends, opening new investigation options for refinement such as adding the influence of object orientation, color, and texture in the feature reconstruction ability of heights, but these are outside the scope of the present research. The poor performance on small individual objects is mainly caused by the continuity constraint of dense matching algorithms. A topic for future research would be to investigate if a combination of different matching strategies can deliver better results on single object heights.

In our investigation, the current findings are valid for Pléiades and WorldView-3 DSMs derived from the tri-stereo images with their specific acquisition geometry. Nevertheless, the acquisition geometry could be changed, but this is only to a very small extend under the control of the user. A higher B/H ratio and implicit convergence angle to the ground will increase the geometric intersection quality, leading to a higher vertical accuracy of the photogrammetric DSM. This will have a positive effect on the single object height signature in the DSM.

From the analyses and investigations performed, the results suggest that the acquisition geometry clearly has an effect on accuracy, but the object height estimation based on automatically derived DSMs primarily depends on object size. Objects of a few pixel in size are hardly mapped in the DSM, whereas, with some dependency on object type, objects of 12 pixels in size are typically mapped with approximately 50% of their real height. To reach 90% of the real height, a minimum object size between 15 and 30 pixel is necessary.

## Figures and Tables

**Table 1:** Acquisition parameters of Pléiades and WorldView-3 data.

Sensor Type & Acquisition Date	View	Acquisition Time (hh:hm:ss.s)	Incidence Angles (°)			Area (km^2^)	B/H Ratio	Convergence Angle (°)
			Across	Along	Overall			
Pléiades 13-06-2017	Forward (F)	10:09:51.5	−2.23	−6.75	6.75	158.73	0.10 (FN)	5.71 (FN)
	Nadir (N)	10:10:03.7	−3.31	−1.13	3.50	158.49	0.11 (NB)	6.30 (NB)
	Backward (B)	10:10:14.0	−5.00	4.95	7.02	158.78	0.21 (FB)	12.0 (FB)
WorldView-3 08-04-2018	Forward (F)	10:22:07.0	7.71	11.00	13.57	100.00	0.20 (FN)	11.52 (FN)
	Nadir (N)	10:22:25.5	7.23	−0.62	7.36	100.00	0.20 (NB)	11.51 (NB)
	Backward (B)	10:22:44.1	6.72	−12.20	13.97	100.00	0.40 (FB)	23.04 (FB)

(FN): Forward-Nadir, (NB): Nadir-Backward and (FB): Forward-Backward image pairs.

**Table 2:** Standard deviation of adjusted tie points (TPs) coordinates.

Sensor Type	No. of TPs/Image	Standard Deviation (m/pixels)					
		Latitude (Along Track)		Longitude (Across Track)		Elevation	
		min	max	min	max	min	max
Pléiades	561/552/582	0.16/0.23	0.33/0.47	0.14/0.20	0.27/0.38	1.49/2.12	3.49/4.98
WorldView-3	556/585/554	0.09/0.30	0.19/0.63	0.08/0.26	0.20/0.66	0.38/1.26	0.74/2.46

**Table 3:** Root mean square error (RMSE) values of ground control point (GCP) and check point (CP) discrepancies.

Sensor Type	No. of GCPs/CPs	RMSE Values (m/pixels)		
		Latitude (Along Track)	Longitude (Across Track)	Elevation
Pléiades	43 GCPs	0.20/0.29	0.19/0.27	0.27/0.39
	50 CPs	0.44/0.63	0.53/0.76	0.85/1.21
WorldView-3	22 GCPs	0.06/0.21	0.08/0.29	0.11/0.37
	50 CPs	0.13/0.43	0.12/0.40	0.28/0.93

**Table 4:** Vertical accuracy assessment of Pléiades and WorldView-3 tri-stereo digital surface models (DSMs).

Sensor Type	No. of GCPs/CPs	*µ*	*σ*	RMSE
Pléiades	43 GCPs	0.07	0.28	0.29
	50 CPs	0.07	0.30	0.31
WorldView-3	22 GCPs	0.03	0.18	0.12
	50 CPs	0.01	0.13	0.13

*µ* and *σ* are the mean and standard deviations. All values are given in meters.
